# The intracellular signalling pathway signature (the signalome) in PBMCs in the presence of a common TRAPS-associated genetic variant, TNFRSF1A p.(Arg121Gln) (legacy p.R92Q) is distinct from normal PBMCs and from other pathogenic variants

**DOI:** 10.1186/1546-0096-13-S1-P19

**Published:** 2015-09-28

**Authors:** A Bybee, O Negm, W Abduljabbar, L Fairclough, H Lachmann, T Lane, I Todd, P Hawkins, P Tighe

**Affiliations:** 1 UCL, National Amyloidosis Centre, London, UK; 2 University, Immunology, Nottingham, UK

## Question

Like other disorders with a strong genetic association, a growing sequence dataset from various autoinflammatory syndrome patients continues to identify many variants of uncertain significance (VUS), i.e. missense and intronic variants or small insertions/deletions, for which the impact on protein function and pathways, and therefore the clinical significance, is unknown. Since it is unclear how these variants are associated with an increased risk of autoinflammatory disease, the clinical management of carriers of VUS is complicated. Therefore, there is a strong demand for reliable tests to rapidly assess the clinical significance of VUS, providing carriers of these variants with the necessary information to make an informed clinical decision and refining treatment by stratified therapy strategies.

In many cases, a novel VUS is not common enough to evaluate its significance. Common variants with apparently variable penetrance can be more accessible as a model to test functional aspects. A missense variant in TNFRSF1A rs41495584 (“p.R92Q”) is the most commonly identified variant associated with TRAPS within our mainly Caucasian UK patient population. The aim of this study was to comprehensively examine the intracellular signalling pathways that are affected by the presence of p.R92Q, in comparison to normal cells and to well-known symptomatic variants such as p.C33Y.

## Methods

PBMCs were collected from patients and healthy volunteers with informed consent. Reverse-phase protein microarray was applied to examine a large number of signalling molecules and inflammatory cytokines, using a feature subset selection process to identify distinctive subsets.

## Results

The resulting p.R92Q patient signatures demonstrated that a particular range of inflammation-associated pathways were dysregulated in p.R92Q variant carriers, grouping carriers together and readily distinguishable from normal PBMCs and other known pathogenic variants.

## Conclusions

The inflammatory signaling pathways activated by the TRAPS-associated variant p.R92Q are distinctive and provide an opportunity to identify a strategy for correlation of genetic findings and functional analysis. This is applicable to straightforward or subtle phenotypes, and common or rare genetic variants not only in TRAPS, but also in other autoinflammatory diseases such as CAPS, FMF, MKD, Blau syndrome and others. This represents an important step towards genetic-bioinformatic disease portraits with statistical and clinical relevance.

